# Bridging traditional and deep learning methods in H&E histological image normalization: a comprehensive review and introducing a novel framework for comparative analyses

**DOI:** 10.1016/j.jare.2025.09.049

**Published:** 2025-10-13

**Authors:** Behnaz Haji Molla Hoseyni, Sevda Imany, Ahmadreza Iranpour, Maryam Mehrabani, Sina Seifouri, Maryam Rafieipour-Jobaneh, Sina Firuzbakht, Ali Masoudi-Nejad

**Affiliations:** a Laboratory of Systems Biology and Bioinformatics (LBB), College of Engineering Science, University of Tehran, Tehran, Iran; b Laboratory of Systems Biology and Bioinformatics (LBB), Department of Bioinformatics, Kish Intl. Campus, University of Tehran, Tehran, Iran; c Department of Computer Engineering, Sharif University of Technology, Tehran, Iran

**Keywords:** Histology images, Normalization, Deep learning, Digital pathology

## Abstract

**Background:**

Histology images are a cornerstone of pathology, which allow automated analysis for disease diagnosis. However, variations in staining and image acquisition processes significantly affect the performance of these algorithms. Histology image normalization is method to achieve uniformity in image color distributions, which will enhance the accuracy and consistency of automated analysis.

**Aim of review:**

This review was conducted with the aim of assessing normalization methods and comparing them in an empirical manner to help researchers choose the most appropriate method for their study. It also aims to assist academics and professionals involved in automated image analysis and digital pathology.

**Key scientific concepts of review:**

This review categorizes normalization techniques into four groups: deep learning-based approaches (e.g., GANs, autoencoders, diffusion models), traditional methods (e.g., deconvolution, histogram matching), hybrid models, and a novel signal processing-based method. It also introduces a new deep learning framework for evaluating normalization strategies and experimentally compares eight state-of-the-art methods on histopathology images. The results highlight the strengths and limitations of each approach, helping researchers and professionals choose suitable methods for their needs. In addition, the review emphasizes the impact of color variation on the accuracy of computer-aided diagnosis (CAD) systems and the importance of preserving biological information during normalization. Finally, it outlines directions for future research, including integrating normalization with data augmentation and exploring information preservation beyond cancer subtype.

## Introduction

In recent years, histopathology images have become an essential tool for diagnosing various diseases, including cancer [1,2]. Histopathology involves the analysis of biopsy samples conducted by a pathologist. These images offer a detailed view of tissue structures and biological compositions. Consequently, the examination of histopathology images is a significant research field aiding in disease diagnosis. Pathologists often diagnose diseases from histopathology images using microscopes and visual analysis of tissue structures. However, this manual process has several challenges. The large number of images that need to be examined, along with the complexity of cell structures, makes the task highly time-consuming and labor-intensive. As a result, there is a growing need for computerized methods that can assist pathologists by reducing their workload and improving the efficiency and accuracy of disease diagnosis [3]. In histopathology images, tissue samples are usually highlighted using tissue staining. One of the most common types of staining is H&E staining in which two types of stains called hematoxylin and eosin are commonly used distinguishing cell nuclei, cytoplasm [4] and etc. This technique is widely preferred because it is relatively simple to perform while also highly effective in providing clear and detailed views of tissue morphology. Hematoxylin stains the nuclei of cells a deep blue or purple color, making it easy to observe their shape and structure, while eosin stains the cytoplasm and extracellular components in varying shades of pink. This information allows pathologists to assess the organization of cells and also identify abnormalities, and pathological changes, such as those seen in cancer [5]. With the advancement of science and the introduction of digital technologies into this field, histopathology images have also begun to be digitized. Initially, digital imaging in histopathology involved capturing static images using cameras, but with the development of whole slide imaging (WSI), it has become possible to digitize entire histology slides at high resolution. Advanced scanners are used to scan histopathological tissue sections and store them as high-resolution digital files as WSI. WSI allows pathologists to navigate, zoom, and analyze tissue samples through a computer interface that replicates the experience of using a traditional light microscope. The widespread use of these digital images has introduced intelligent computer systems for automatic disease diagnosis, particularly in cancer detection, aiding specialists in this field in diagnosis and treatment. These systems enhance accuracy by providing quantitative analysis and reducing reliance on subjective assessments. Furthermore, artificial intelligence (AI) has the potential to uncover insights that may be complex or traditionally overlooked in conventional analysis. AI can also speed up clinical analysis by automating manual tasks. In some cases, deep learning models perform as well as or even better than human experts. A study trained a convolutional neural network (CNN) using 12,378 open-source dermoscopic images. In a head-to-head comparison involving 100 images, the CNN outperformed 136 of 157 dermatologists across various experience levels in classifying dermoscopic melanoma images [6]. Another study [7] demonstrated that a deep learning model outperformed 11 pathologists in classifying histopathological images of melanoma. The AI system achieved higher accuracy in distinguishing between malignant and benign samples, showcasing its potential as a diagnostic aid in pathology.

The proliferation of these systems has posed a new challenge for researchers, pathologists, physicians, and other users of these systems. Histopathology images vary due to factors such as color fading of used stains (hematoxylin and eosin), manufacturer reactivity, different microscopes, concentration of used colors, varying staining times, and the imaging using different scanners [8]. Such variations can unintentionally cause diagnostic algorithms to rely on irrelevant, institution-specific features rather than relevant biological patterns, thereby reducing their ability to generalize across datasets from different hospitals and institutions [9]. This diversity can significantly impact the intelligent automatic diagnosis systems that use these images, reducing their accuracy noticeably [10]. It causes the algorithms used in these systems to be trained on different images and may not perform well in the process of diagnosing cancer or various diseases. Color normalization stands as a pivotal process aimed at alleviating the undesirable color variations that frequently occur among analogous biological components within a single image or across images in a dataset. Its fundamental goal is to uphold the inherent structural and histological information contained within these images while establishing a standardized color representation. To address this issue, various normalization methods have been introduced in recent years [11,12], including methods by Macenko [13], Reinhard [14], and Vahadane [15]. These methods try to mitigate color disparities among biological components, thereby ensuring more consistent and dependable analyses, all while preserving the indispensable structural and histological details embedded within the images. Some of these methods use statistical and mathematical approaches, while others utilize deep learning and various artificial intelligence-based methods. However, it is important to consider that each of these methods has its advantages, disadvantages, and specific limitations. Some focus solely on structural alterations, while others concentrate only on color and patches for normalization. Yet others may consider different ratios to assess their collective significance. Consequently, our primary contributions to this article can be summarized as follows:

We first propose a new categorization of normalization methods for histopathology images, covering a wide range of approaches such as deep learning-based models (including GANs, autoencoders, and diffusion models), mathematical optimization techniques (e.g., global and local transformation-based methods), signal processing approaches, and hybrid frameworks. This categorization enables a more structured understanding of the field and facilitates method-specific investigations.

Next, we provide a comprehensive evaluation of eight representative normalization methods, selected based on their diversity in methodology and code availability. These methods are compared using multiple quantitative metrics to assess their effectiveness in addressing staining variations.

Finally, we introduce a novel evaluation framework based on deep learning models, which enables assessing normalization performance in terms of both biological information preservation and staining variation removal. This deep learning-based assessment allows for a more application-relevant and biologically meaningful comparison of normalization methods.

## Materials and methods

This section outlines the search terms utilized and academic search engines employed to retrieve relevant articles. We provide an overview of the datasets used to perform the selected normalization methods. Additionally, we explain the quantitative metrics utilized and present our proposed approach for comparing different normalization methods.

### Search strategy

A comprehensive search was conducted using Google Scholar and PubMed, limited to articles published between 2000 and May 2025. The search query included terms related to normalization and histopathology images in the title, as follows:

(((“normaliz*”) OR (“Color Normalization”) OR (“Standardization”)) AND ((“pathology image”) OR (“WSI”) OR (“whole slide image”) OR (“histopathology”) OR (“pathology”) OR (“digital pathology”) OR (“pathomics”) OR (“he”) OR (“H&E”) OR (“histological”) OR (“histopathol*”) OR (“histology”) OR (“digital pathology”))) OR ((“Histopathology Image Analysis”) OR (“Stain Normalization”) OR (“Stain Estimation”)).

The selection of papers followed a two-step evaluation process. Initially, papers were assessed based on titles, and then abstracts were reviewed. Additional papers were included by utilizing references from the identified articles. This evaluation process resulted in a final collection of 48 selected papers for further analysis.

### Evaluation metrics

This section describes the conventional metrics commonly used for evaluating normalization methods. Additionally, we introduce our novel approach based on deep learning techniques to compare and assess the performance of these methods.

#### Structural similarity index measure

The structural similarity index measure (SSIM) metric in this study evaluates the structural similarity between the pre-normalization image (S) and the post-normalization image (I) [16].

$$SSIM\left(S,I\right)={\left[l\left(S,I\right)\right]}^{\alpha }.{\left[c\left(S,I\right)\right]}^{\beta }.{\left[s\left(S,I\right)\right]}^{\gamma }$$

where *l* represents illumination, *c* represents contrast, and *s* represents the structure similarity between the S and I images.

Illumination is measured by capturing the mean intensity of the pixels in the image I. This provides insight into the overall brightness level of the image.

$$\mu_{I}=\frac{1}{N}\sum_{i=1}^{N}I_{i}$$

N is the number of pixels, and the function l measures illumination similarity between 2 images as:

$$l\left(S,I\right)=\frac{2\mu_{S}\mu_{I}+C_{1}}{\mu_{S}^{2}+\mu_{I}^{2}+C_{1}}$$

$C_{1}$ is constant to avoid instability.

Contrast quantifies the variation in pixel intensities and the range of shades present. Estimation of contrast of image S was defined as the standard deviation of intensity of pixels:

$$\sigma_{S}={\left(\frac{1}{N-1}\sum_{i=1}^{N}{\left(S_{i}-\mu_{S}\right)}^{2}\right)}^{\frac{1}{2}}$$

and similarity of contrast of 2 images was computed as:

$$c\left(S,I\right)=\frac{2\sigma_{S}\sigma_{I}+C_{2}}{\sigma_{S}^{2}\sigma_{I}^{2}+C_{2}}$$

as mentioned before $C_{2}$ is constant.

At final, structural information of the image I obtained as:

$$\frac{I-\mu_{I}}{\sigma_{I}}$$

Lastly, the structure similarity component assesses the likeness of structural patterns between the S and I images. To measure structural similarity, we compare the correlation or dot product between the structure of the two images. This is equivalent of the correlation between S and I, so structural similarity was defined as:

$$s\left(S,I\right)=\frac{\sigma_{\mathrm{SI}}+C_{3}}{\sigma_{S}\sigma_{I}+C_{3}}$$

where $C_{3}$ is constant and $\sigma_{\mathrm{SI}}$ is the correlation coefficient of S and I and formulated as:

$$\sigma_{\mathrm{SI}}=\frac{1}{N-1}\sum_{i=1}^{N}\left(S_{i}-\mu_{S}\right)\left(I_{i}-\mu_{I}\right)$$

In the formulation of SSIM, the parameters α, β, and γ are used to adjust the relative importance of each component (illumination, contrast, and structure) in the overall similarity calculation. However, in the original article, for simplicity, these parameters were set to fixed values. Specifically, α, β, and γ were all considered to be 1, and it was assumed that $C_{3}=\frac{C_{2}}{2}$.

#### Multi-scale structural similarity

Since the evaluation of image quality depends on viewing conditions, including screen resolution or viewing distance, different scales have been used in this metric to evaluate the quality of images. The multi-scale structural similarity (MS-SSIM) metric compares images across multiple scales, utilizing a similar SSIM calculation[17]. The original images are considered at scale 1, and then a low-pass filter is applied followed by downsampling the image by a factor of 2 at each step.

$$\mathrm{MS}-SSIM\left(S,I\right)={\left[l\left(S,I\right)\right]}^{\alpha_{M}}\Pi_{j=1}^{M}{\left[c_{j}\left(S,I\right)\right]}^{\beta_{j}}{\left[s_{j}\left(S,I\right)\right]}^{\gamma_{j}}$$

where j is the index of scaled images, and α,β,γ represent the parameters involved in the calculation. It is important to note that the similarity of illumination is only computed at the Mth scale.

#### Peak-signal-to-noise ratio

PSNR stands for Peak Signal-to-Noise Ratio, and it is a metric commonly used to evaluate the quality of an image or a video [18]. The PSNR provides a quantitative assessment of the quality of normalized images by comparing pixel-wise differences between the original and normalized images. A higher PSNR value indicates better preservation of image information during the normalization process. If S is the image before perturbation (in this field normalization) and I is the image after normalization PSNR was defined as:

$$PSNR\left(S,I\right)=10\log_{10}\left(\frac{255^{2}}{MSE\left(S,I\right)}\right)$$

where MSE is the mean square of the pixel-wise distance between S and I.

#### The normalized median intensity

The normalized median intensity (NMI) is employed as a metric to measure and compare the extent of intensity variation within a population of images resulting from different Normalization methods[19]. This was defined as:

$$NMI\left(I\right)=\frac{{\mathrm{Median}}_{i\in I}\left\{U\left(i\right)\right\}}{{\mathrm{Max}}_{i\in I}\left\{U\left(i\right)\right\}}$$

U is the average of R, G, and B channels, i is pixel, and I indicates image. The standard deviation and coefficient of the variation of the NMI values were computed for images of all resources, before and after stain normalization. The smaller these values are, the more similar the images.

#### Feature similarity index

The feature similarity index (FSIM) is a metric designed to measure the structural similarity between two images. It is capable of assessing the structural similarity in both color and grayscale images[20]. In grayscale images, the measurement of image similarity combines two measures: phase congruency (PC) and gradient magnitude (GM). Phase Congruency is a dimensionless measure that captures the significance of local structures. It assumes that features are perceived at the points where the Fourier components reach their maximum. This method is commonly used for edge detection and is invariant to variations in illumination and contrast.

On the other hand, GM calculates the gradients of images using convolution masks. By convolving the masks with the images, the horizontal and vertical gradients $G_{x}$ and $G_{y}$ are obtained. The gradient magnitude that represents the overall strength of the gradients in the image, computed as

$$G=\sqrt{G_{x}^{2}+G_{y}^{2}}$$

The similarity of PC between images S and I at local location x can be defined as follows:

$$S_{\mathrm{pc}}\left(x\right)=\frac{2{\mathrm{PC}}_{s}\left(x\right){\mathrm{PC}}_{I}\left(x\right)+C_{1}}{{\mathrm{PC}}_{S}^{2}\left(x\right)+{\mathrm{PC}}_{I}^{2}\left(x\right)+C_{1}}$$

where $C_{1}$ is the positive constant value to avoid instability, and ${\mathrm{PC}}_{s}$ is the Phase Congruency of image S. In similar, the gradient magnitude similarity between S and I was defined as follows:

$$S_{G}\left(x\right)=\frac{2G_{s}\left(x\right)G_{I}\left(x\right)+C_{2}}{G_{S}^{2}\left(x\right)+G_{I}^{2}\left(x\right)+C_{2}}$$

where G represents the gradient magnitude and $C_{2}$ is a positive constant value. The combination of these two measurements at local location x is defined as follows:

$$S_{L}\left(x\right)={\left[S_{\mathrm{pc}}\left(x\right)\right]}^{\alpha }.{\left[S_{G}\left(x\right)\right]}^{\beta }$$

Since different locations in images, such as edge locations, have varying contributions to the perception of the human visual system compared to smoother areas, it is necessary to consider the distinct contributions of different locations when calculating the final similarity score. This is due to the fact that the PC measure reflects perceptually significant structures. Therefore, to account for these factors, the FSIM metric for grayscale images was defined as:

$$FSIM\left(S,I\right)=\frac{\sum_{x\in \Omega }S_{L}\left(x\right).{\mathrm{PC}}_{m}\left(x\right)}{\sum_{x\in \Omega }{\mathrm{PC}}_{m}\left(x\right)}$$

x represents the location of the image, and Ω represents the image and

$${\mathrm{PC}}_{m}\left(x\right)=max\left({\mathrm{PC}}_{I}\left(x\right),{\mathrm{PC}}_{S}\left(x\right)\right)$$

In the case of color images, the images are first converted from RGB to YIQ color space $S_{L},{\mathrm{PC}}_{m}$ performed on the Y channel, which represents the illumination information of the image and on I,Q channel similarity was computed as:

$$S_{I}\left(x\right)=\frac{2I_{I}\left(x\right).I_{S}\left(x\right)+C_{3}}{I_{I}^{2}\left(x\right)+I_{S}^{2}\left(x\right)+C_{3}},S_{Q}\left(x\right)=\frac{2Q_{I}\left(x\right).Q_{S}\left(x\right)+C_{4}}{Q_{I}^{2}\left(x\right)+Q_{S}^{2}\left(x\right)+C_{4}}$$

The combination of the $S_{I}$ and $S_{Q}$ is calculated as:

$$S_{c}\left(x\right)=S_{I}\left(x\right).S_{Q}\left(x\right)$$

The final FSIM score for color images is obtained as:

$${\mathrm{FSIM}}_{c}\left(S,I\right)=\frac{\sum_{x\in \Omega }S_{L}\left(x\right).{\left[S_{c}\left(x\right)\right]}^{\gamma }{\mathrm{PC}}_{m}\left(x\right)}{\sum_{x\in \Omega }{\mathrm{PC}}_{m}\left(x\right)}$$

#### Learned perceptual image patch similarity

The learned perceptual image patch similarity (LPIPS) measurement employs a pre-trained deep learning model, such as VGG or AlexNet [21]. The activations of L convolutional layers are extracted for both the source (S) and target (I) images. The distance between these activations is then computed using the formula:

$$d\left(S,I\right)=\sum_{l}\frac{1}{H_{l}W_{l}}\sum_{h,w}{\|w_{l}.\left({\hat{S}}_{\mathrm{hw}}^{l}-I_{\mathrm{hw}}^{l}\right)\|}_{2}^{2}$$

where $S_{\mathrm{hw}}^{l},I_{\mathrm{hw}}^{l}\in R^{H_{l}\times W_{l}\times C_{l}}$ represent the activations of layer l for images S and I, respectively. $w_{l}\in R^{C_{l}}$ denotes the parameters used to scale the activations channel-wise. By considering w = 1, the computed distance is converted to cosine distance. This metric has been calibrated with human perceptual judgment data, giving rise to the LPIPS metric.

#### Deep learning-based evaluation approach

In order to assess the effectiveness of stain normalization methods, we propose a novel approach using deep learning models. Our evaluation framework involves a dataset comprising images acquired from two distinct scanners. At first, various normalization techniques were employed in this data, and then a deep learning model was trained to classify scanner labels on the normalized images. From our perspective, if a particular normalization method results in a lower model performance to classify scanners, we can infer that the method has effectively eliminated the distinctive characteristics associated with different scanners. As a consequence, the model becomes unable to differentiate between images originating from different scanners.

Additionally, we have a separate dataset consisting of images with assigned subtype cancer labels. In this case, we will normalize the images using the same normalization methods and subsequently train another deep learning model with the aim of predicting the cancer subtype of the image. If the model achieves high accuracy in this dataset, we can infer that the chosen normalization method has successfully preserved the essential biological information present in the images.

### Dataset

Three datasets have been used for the comprehensive analysis of this study.

1. SCAN Dataset [22]

The H&E images of this dataset are obtained from 5 tissues (adrenal, breast, colon, liver, and prostate) with three magnifications of 10x, 20x, and 40x. this study images with 10x and 20x magnifications were used.

2. MITOS-ATYPIA-14 Dataset [23]

The images in this dataset were obtained from H&E staining of breast tissue and were imaged with two Aperio Scanscope XT and Hamamatsu Nanozoomer 2.0-HT scanners. These images are available in two magnifications of 20x and 40x.

3. TCGA-BRCA Dataset [24]

We selected a subset of paraffin embedding H&E images of TCGA-BRCA. All images are from the breast but are from six histopathology subtypes of breast cancer. We included an approximately equal number of WSIs from each subtype. Overly, there are an equal number of whole slide images of each cancer subtype within this selected subset.

Fig. 2, Fig. 3, Fig. 4, Fig. 5 were generated using the ggplot2 [25] package in R.

## Comprehensive review

**Fig. 1:**
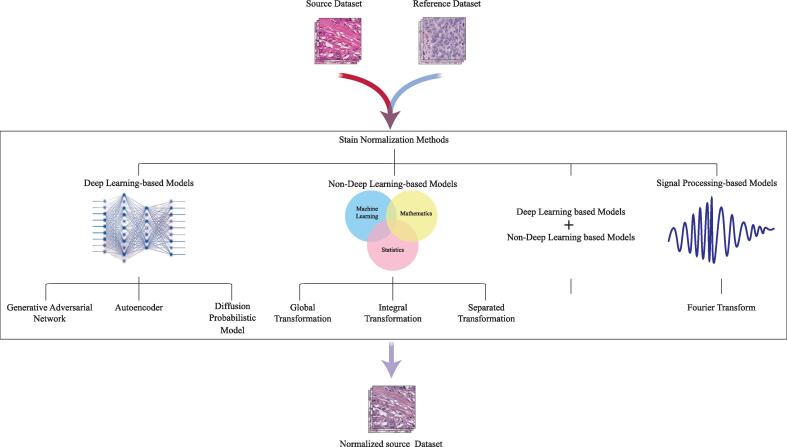
Overview of the categories of normalization methods.

Color variation in histopathology images affects the accuracy of computer-aided diagnosis (CAD) systems [26]. Existing methods to overcome this problem and normalize images can be divided into four groups: 1) Deep learning-based solutions, 2) Non-deep learning solutions, 3) Hybrid models and 4) signal processing-based (Fig. 1). The choice between these approaches depends on factors such as the available data, computational resources, and the specific requirements of the application. Non-deep learning solutions are generally computationally efficient and faster, but on the other hand, deep learning-based solutions can capture more complex color variations. However, they require large amounts of data for training and can be computationally intensive.

**Fig. 2:**
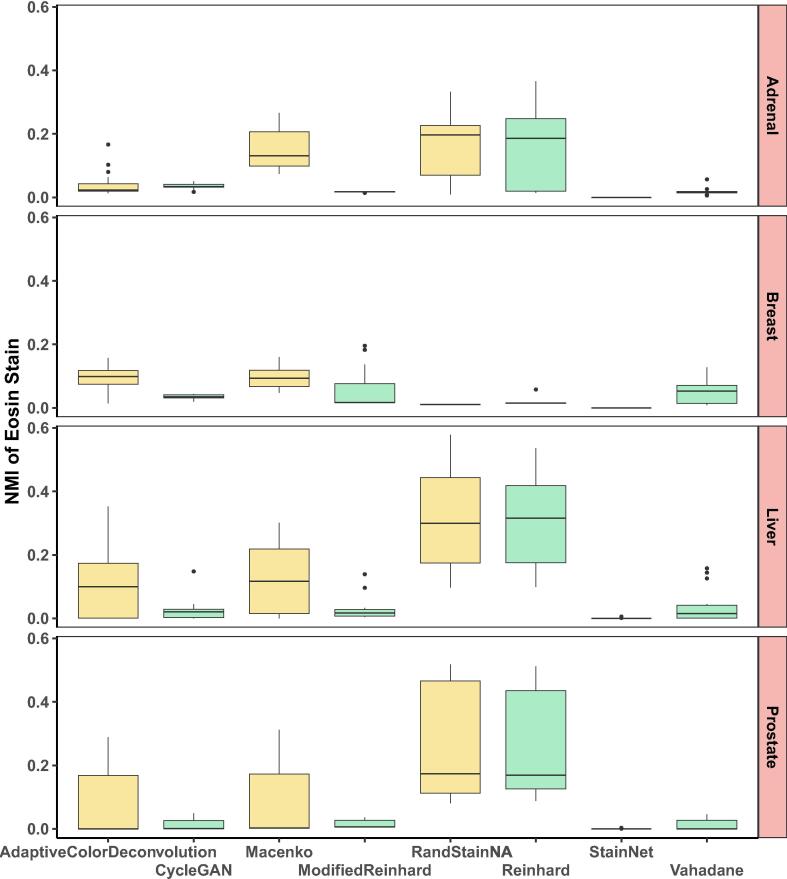
NMI values of eosin stain of images at resolution 10x.

**Fig. 3:**
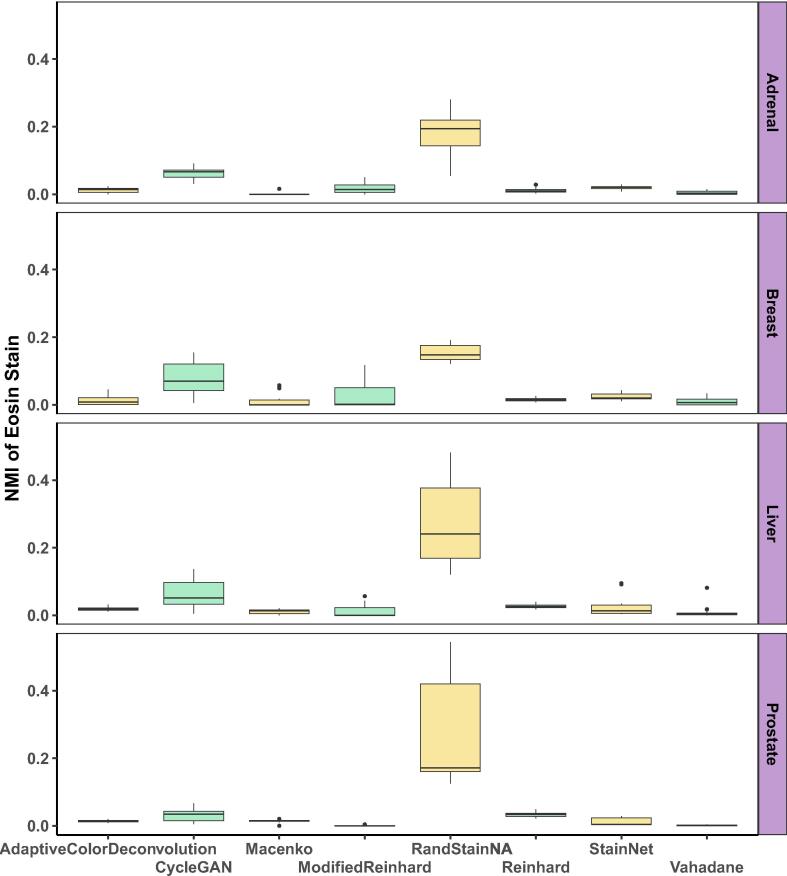
NMI values of hematoxylin stain of images at resolution 10x.

**Fig. 4:**
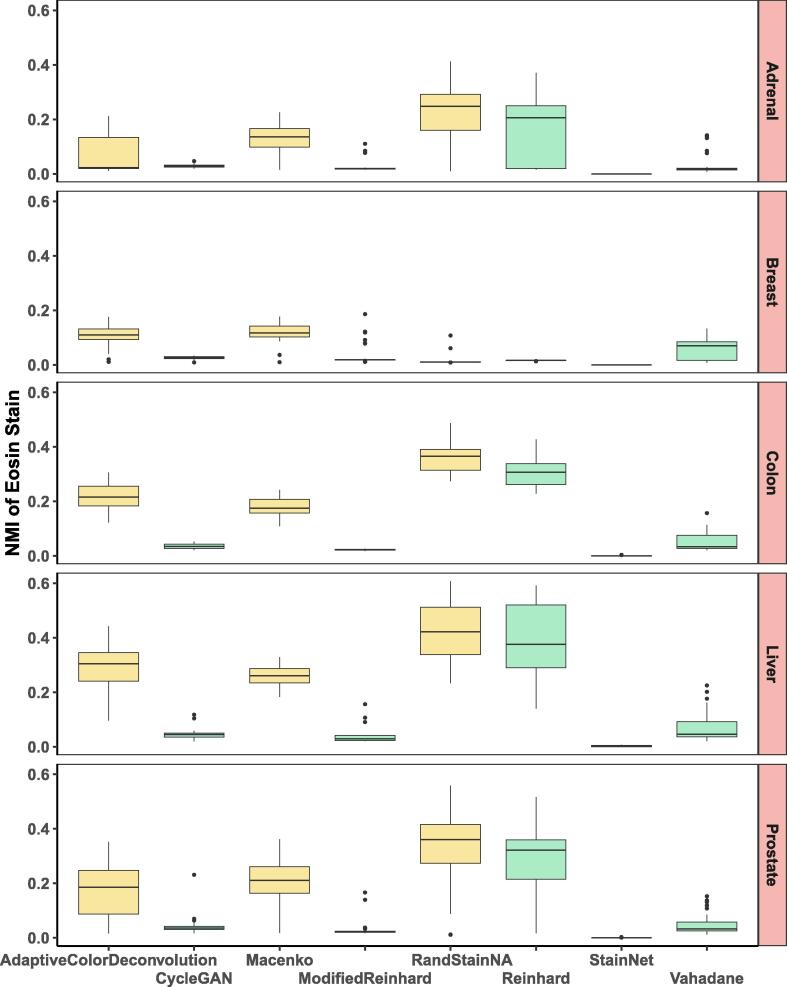
NMI values of eosin stain of images at resolution 20x.

**Fig. 5:**
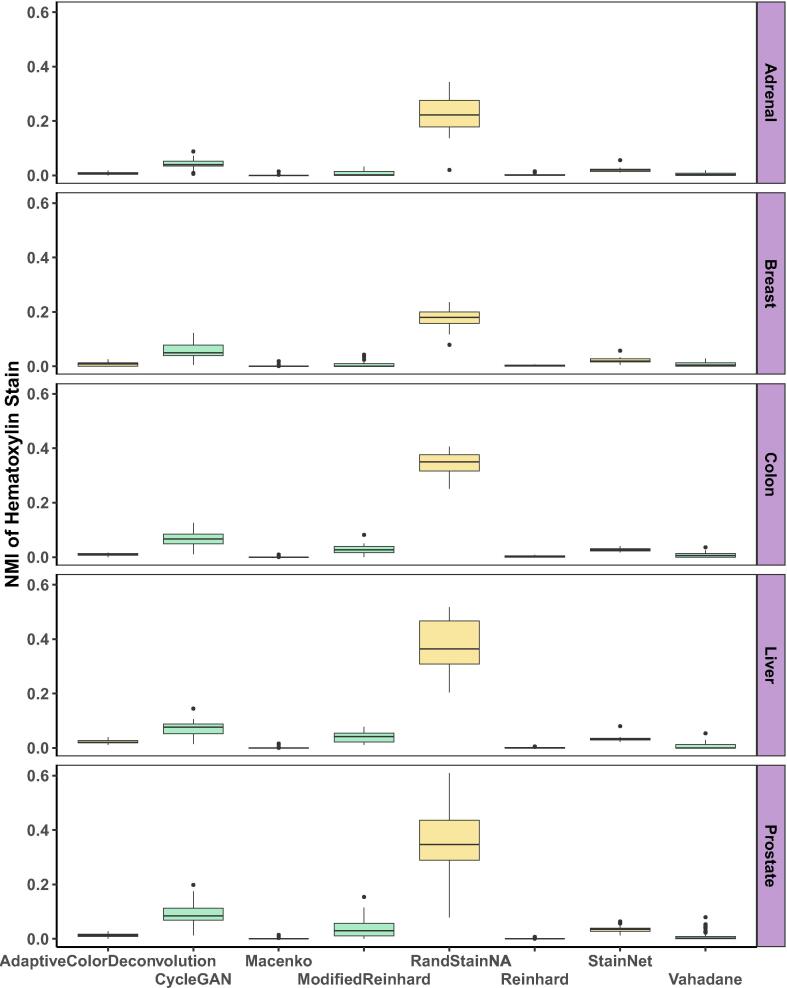
NMI values of hematoxylin stain of images at resolution 20x.

### Deep learning-based solutions

With many advances in the field of deep learning, researchers have provided various methods for color normalization using deep learning. These approaches typically involve training deep neural networks to learn image transformations that can normalize color variations.

The deep learning-based solutions can be categorized into three groups: (1) Generative Adversarial Networks (GANs), (2) approaches using Auto-Encoder, and (3) Diffusion Probabilistic Models.

#### Generative adversarial networks (GANs)

Generative Adversarial Networks (GANs) were first introduced in 2014 [27]. The paper proposed the GAN framework and introduced the adversarial training approach, where a generator network and a discriminator network are trained simultaneously in a min–max game setup with the aim of generating fake data similar to real data. Since its introduction, GANs have become a fundamental concept in the field of deep learning and have been widely employed for various tasks. In stain normalization, GANs have shown promising results in addressing color and stain variations by generating synthetic images that resemble a target distribution.

CycleGAN [28] is one of the most well-known unpaired image-to-image translation models and has been used for histology image normalization [29]. An approach for stain normalization of histology images was introduced in [30]. StainGAN is a stain normalization method that uses CycleGAN [28] to standardize the color of histology images. Unlike traditional approaches, it does not require a reference slide. StainGAN consists of generator and discriminator networks that map images between different scanners. The generators aim to match the target domain, while the discriminators distinguish real from generated images. A cycle-consistency constraint ensures the preservation of tissue structure. StainGAN heavily relies on the use of GANs, which can be challenging to train and require a significant amount of computational resources.

On the contrary, StainNet [31] is a stain normalization method that is more than 40 times faster than StainGAN and utilizes a fully 1x1 convolutional neural network trained through distillation learning, which makes it very fast and robust. The method incorporates StainGAN as a teacher network to generate normalized images, which are then used to train StainNet. By minimizing the difference between StainNet's output and StainGAN's normalized images, StainNet adjusts color values in a pixel-by-pixel manner, achieving effective stain normalization while preserving source image information. But, because of its simple network structure, StainNet is not able to learn complex stain normalizations.

In the mentioned models, one dataset was selected as the source and one dataset as the reference, and an attempt was made to map the source dataset to the reference dataset space with the generator of the model. Same as StainGAN, a method based on the CycleGAN [28] approach for unpaired image-to-image translation to solve the stain normalization problems was proposed in [32]. Transitive adversarial networks (TAN) avoid the need to manually select a reference template slide by allowing bidirectional mapping between two domains. Cycle-consistency constraint ensures that the generated images preserve the original structure. The generator architecture in the TAN method, called Trans-Net, is specifically designed to produce more accurate color transfer by incorporating information before and after sampling.

The normalization models that were reviewed so far are based on CycleGAN [28]; although some of them solve the problem of selecting a reference, they also have some limitations. CycleGAN [28] methods require memory resources and they typically involve a significant amount of training time. StainNorm [33], which employs CycleGAN, reduces the source-complexity training time by up to forty times by employing a lightweight 1 × 1 convolutional layer and a different training procedure. The training procedure is performed in several phases and in a streamlined manner. First, the generators in this model learn the identity function. Then, curriculum learning [34] is applied by gradually adding color jitter and increasing the complexity and intensity of the changes to the source images. Additionally, the Background Output Regularization stage improves the model by producing a pure background image using Gaussian noise and normalizing it to match the target background. Also, a method named StainCUT was proposed in [35] to address computational resource-related disadvantages. The proposed method does not rely on the CycleGAN [28] approach and it is based on GAN as well as contrastive loss. The used generator of this model contains encoder and the decoder part and the basis of StainCUT is based on another study that showed that the encoder includes “content” and the decoder includes “style” [36]. A contrastive learning objective function trains representations by ensuring that similar elements are aligned while dissimilar elements are differentiated. They used the contrastive function on the information embedded by several layers of encoders for the source data and the data generated by the generator. To preserve the content between the source data and its mapped data, Contrastive Loss considers that the embedded of same locations are similar and the different locations have different embedded.

Most existing models transform images between domains without explicitly disentangling color appearance (e.g., hematoxylin and eosin hues) and stain density (e.g., cellular structures), which can hinder their adaptability and interpretability. To address this limitation, TredMiL [37], a deep latent model based on a Least Squares GAN, was proposed. It disentangles color appearance and stain density into independent latent codes using a generator that includes two separate encoders. These codes are then combined and passed to a decoder to reconstruct the image. A truncated Gaussian mixture prior is applied to the color appearance latent space to reduce sensitivity to outliers and better manage overlapping stains.

Another approach is the stain-variability normalization (SVN) method introduced in [38], which aims to re-stain H&E images to match IHC images by utilizing GANs. SVN incorporates three essential losses: the structural loss (Lr) using the Difference of Gaussian (DoG) filter, the strain-variation loss (Lc) for aligning staining patterns, and the adversarial loss (La) to ensure realistic synthesized images. Through the combination of these losses, SVN achieves accurate stain normalization, effectively addressing staining variabilities and enhancing the quality and consistency of histological image analysis. Although this model showed good performance, its potential weakness is its need for two types of H&E and IHC images.

As previously mentioned, one of the reasons for using normalization methods is to improve deep learning models for predicting biological information on unseen data, and studies have shown that using normalization methods can improve the performance of models [39]. In a study, an attempt was made to use biological information contained in images to improve normalization performance, just as normalization is used to improve biological task prediction. The researchers used a CycleGAN model in which the generator consisted of an autoencoder model comprising an encoder to encode the input image into a bottleneck and two decoders—one for image styling or normalization and the other for nuclei cell segmentation purposes. A discriminator, similar to those in previous models, was employed to distinguish between real and generated normalized images. Due to the lack of data, pixel-wise annotation was used to train the teacher-student model for segmentation. However, regardless of the model architecture, performing the normalization and segmentation tasks simultaneously improves the performance of normalization [40].

The researchers designed the DSTGAN [41] model based on GANs, which employs a two-stage stain normalization strategy—using supervised learning on the target domain and semi-supervised learning on the source domain—to extract as much information as possible from the entire dataset. In addition to convolutional layers, the generator structure of this model also uses the Swin Transformer as a backbone so that the model can simultaneously identify and correctly preserve local features and long-range dependencies in histological images. The model includes a generator and two discriminators: one to detect whether the color of the image matches the target domain, and the other to determine whether the texture is realistic. The first discriminator distinguishes between real images from the target domain and fake images generated by the generator. The second discriminator evaluates the authenticity of the texture by distinguishing among images from the source/target domain and the generated images. Generator input is gray-scale image. Ground truth result for images from target domain are available, so learning of this domain was supervised manner. Employing on source images include two step, at first as same as images from target, image convert to gray-scale and fed to model and colored images was generated then this image convert two gray-scale and fed to the model again, enabling semi-supervised learning using pseudo-labels obtained from the previous step. Although DSTGAN effectively generates high-quality stain-normalized images, it faces challenges such as high computational cost, training instability, and limited dataset size.

The mentioned models have two problems. In these models, normalization is done between two datasets, one as a source and one as a reference. In this case, If, for example, we have pathology images from several laboratories, an algorithm must be executed several times between different pairs, which is time-consuming. The second problem is related to data privacy. To train these models, both source and target datasets must be available. In the rest of this section, some articles that address these challenges.

To address the first limitation, the concept of *meta*-domain was presented in [42]. In this study unpaired image-to-image translation (UI2IT) models were used to stain color normalization. Instead of training a GAN between each pair of data domains, they used a *meta*-domain composed of data from various laboratories. This *meta*-domain (M) served as the source domain during training for stain color normalization. By approximating the mapping between the *meta*-domain and the target domain, they were able to perform stain color normalization without the need for training multiple GANs. In the test phase, they found that applying a double normalization, which performs *meta*-domain mapping on both source and target datasets, improved the results. It shows that the *meta*-domain mapping (GMT) of the source dataset (S) was more similar to the GMT of the target dataset (T) than to the target dataset itself. In another study, MultiPathGAN [43] used a single generator and a single discriminator to translate images between multiple domains. Similar to previous models, the generator is responsible for translating images from first domain to another. In addition to the image, the input to this model also includes a randomly generated target label that specifies the domain to which the input image should be translated. The discriminator, in addition to determining whether the input is real or fake as in a standard GAN, also predicts the domain class. The image generated by the generator is then passed back to the generator, this time with the domain label of the original image, and the distance between the original and reconstructed image is employed in the final loss function of the model. Another notable point that these researchers considered in their study is the emphasis on preserving semantic information between the original image and the normalized image, or the image translated into another domain. They obtained representations of the two images using a pre-trained classification model and incorporated the difference between these two representations into the loss function.

Another study that addressed the limitation due to the multi-domain, employed CycleGAN to colorize grayscale images into the reference domain. By converting RGB images to the 3-channel grayscale space and then recoloring them into the target color space, the input space of the model is reduced, thereby simplifying the problem. The researchers applied augmentation before converting the color images to grayscale, introducing variation into the input with the aim of improving generalization to unseen data domains. Since the input to the two generators in this study is a grayscale image and the output is a color image the standard identity loss was no longer applicable. Instead, a new domain-faithful reconstruction loss was introduced to ensure the consistency between the reconstructed image and its original unaugmented version [44].

The problem of privacy and security of collecting training datasets from multiple institutes was addressed in [45] through the introducing a Federated Learning System. Their central innovation is the introduction of a Decentralized conditional generative adversarial network (cGAN). A global shared generator and multiple client-specific discriminators are employed, allowing for the adaptive generation of a stain style interpolated from a collection of styles. Training occurs in a data-private federated learning paradigm, aiming to optimize a weighted average adversarial loss with assistance from multiple private discriminators. Additionally, the L1-norm between original and normalized features preserves the pattern, and a temporal self-distillation loss was used to stabilize generator training. This loss function aims to address challenges in stain normalization, and promote adaptability, pattern preservation, and stable convergence.

#### Autoencoders

Autoencoders have been widely utilized in the field of stain normalization as a powerful deep learning technique. Autoencoders are neural network models designed to reconstruct their input data, learning an efficient representation of the data in the process. In the context of stain normalization, autoencoders can be used to learn the underlying structure and variability within images affected by different staining protocols.

The method in [46] called StaNoSA that employs sparse autoencoders to learn filters from randomly selected patches of a template image. These learned filters are then utilized to generate feature space representations for both the template and the source image. Subsequently, K-means clustering is applied to the filter responses. Finally, a color standardization process is performed for each cluster using histogram shifting, and the source image will be standardized to the template image.

In a completely different approach, several autoencoder-based methods have utilized augmentation techniques and augmented data to capture the biological structure of the data. The approach in [47,48,49] proposed similar solutions in this area. They employed HSV augmentation to generate images with diverse styles. Another study aimed to normalize images obtained from different scanners and microscopes by restoring distorted images to their original quality using a deep learning-based autoencoder normalizer. To simulate distortions, original images were manipulated by overlaying 12 predefined RGB colors, combined with various levels of brightness, contrast, and saturation, to generate intracellular and/or background distortions. All manipulations were performed within the RGB color space [50].

These methods trained their networks using extensively augmented or distorted H&E images, enabling them to effectively reconstruct the original appearance of the images while mitigating artificially introduced color variations. The network architecture employed in these approaches consists of convolution and deconvolution layers in the encoder and decoder parts with skip connections that allow information to flow between corresponding encoder and decoder layers.

#### Diffusion probabilistic models

Diffusion Probabilistic Models (DPMs) [51], or simply diffusion models, are a class of generative models that have gained significant attention in recent years due to their strong performance in generating high-quality data, specifically in image generation. Diffusion models operate through a two-stage process: a forward (diffusion) process, where noise is gradually added to the input data until it is transformed into pure noise, and a reverse (denoising) process, where a neural network learns to iteratively reverse the predefined noising process with the aim of removing the noise and reconstructing data samples that resemble the original distribution. Unlike GANs, which are trained via an adversarial process that may lead to mode collapse or convergence issues, diffusion models rely on a simple loss function, resulting in a more stable and easier training process. To overcome the challenge of slow sampling inherent in traditional diffusion models, score-based diffusion models, also known as score-based generative models [52], were introduced as a more flexible and efficient alternative. Rather than learning to directly reverse the noise process, these models estimate the score function, which represents the gradient of the log-probability density of the data distribution at different noise levels. This score function is then used to guide the generation of new samples through stochastic processes such as Langevin dynamics or stochastic differential equations (SDEs), resulting in faster and often more accurate sample synthesis.

A study was conducted based on a diffusion model, beginning with the application of sparse non-negative matrix factorization (SNMF) to decompose histopathological images into individual stain components such as eosin and hematoxylin, enabling precise and structure-preserving normalization. A score-based diffusion model is then employed to guide the denoising and reconstruction process, resulting in images with normalized stain appearance. The approach uses an overlapping moving window patch strategy to further enhance consistency across whole-slide images, processing images in overlapping sections to reduce grid artifacts and maintain spatial coherence [53].

### Non-deep learning solutions

In addition to deep learning approaches, non-deep learning methods have also been explored to address color variation. These methods often involve techniques such as histogram equalization, color matching, and other color deconvolution methods. Based on these approaches, non-deep learning solutions can be classified into three categories: 1) global histogram matching methods, 2) integral transformation-based methods, and 3) separated transformation methods. These categories encompass different strategies for addressing color variation in image normalization.

#### Global approaches

Within this category, normalization methods diverge from the practice of segregating distinct stains or categorizing diverse tissues. Instead, their approach is characterized by a global application, wherein the methods are uniformly applied across the entirety of the image. In other words, these methods employ a consistent normalization technique on each segment of the image, disregarding the need for individualized treatment based on specific stains or tissue classifications. This global methodology seeks to ensure a standardized color representation across the entire histopathology slide, aiming for comprehensive normalization without differentiation between various regions or components.

One of the oldest and most well-known methods in this category is Reinhard's method [14]. It begins by converting the RGB image to the lαβ color space. By utilizing the mean and standard deviation of each axis, the method transforms the source image to align its color statistics with the target image. Finally, the transformed image is converted back to the RGB color space. The RandastainNA [54] model also performs a similar method in the LAB color space with the difference that they also do stain augmentation. A modified version of Reinhard's method [55] was also proposed to address two limitations observed in the original approach: loss of background luminance and poor contrast. Similar to the original method, the modified version performs the transformation in the lαβ color space and converts it back to RGB. However, the transformation differs as it incorporates a parameter based on contrast differences between the source and target images. This parameter enhances the transformation process and effectively resolves the limitations mentioned.

RandStainNA++ [56] is an improved version of RandStainNA [54], introducing three key enhancements. First, instead of using a single reference image for normalization, it derives statistical information (mean and standard deviation) from the entire reference dataset to generate a random virtual template for each training image. These templates are sampled in different color spaces (LAB, HED, and HSV) to ensure greater diversity in stain style augmentation. Second, recognizing that foreground and background regions exhibit different staining characteristics, the method applies separate normalization templates to each region, resulting in more realistic and spatially aware color transformations. Third, a self-distillation strategy is employed during training: different versions of the same image—each normalized with a different random template and color space—are encouraged to produce consistent predictions. This consistency loss helps the model learn stain-invariant representations. The normalized images are used to train deep learning models for classification and segmentation tasks on histopathology datasets. Each image is assigned a unique virtual template that changes in every training epoch, allowing the model to learn from diverse stain variations.

In addition to other normalization techniques, transforming images into grayscale is a commonly used method [57]. However, some approaches employ more sophisticated forms of grayscale transformation. For instance, the approach in [58] proposed a quantile normalization technique that includes a color map that maps the intensity of pixels in source images to the intensity of target images. First, they ranked all pixels of both the source and target images, then mapped ith pixel of the source to the corresponding ith pixel of the target. Because of variation in morphology, different peaks in histograms of images; thus, to prevent distortion and maintain morphology, they used quantiles of the histogram of pixel intensity to map. The approach in [59] perform same method but considering 64 different color levels with a self-organizing map, where is a nonlinear algorithm.

The performance of these algorithms is not stable if the tissue composition is different between the reference and the source. Therefore, in an approach, researchers have first tried to obtain several pairs of similar patches in the reference and the source, and then perform normalization using a transformation on rotated and scaled at the center of the white color RGB vector in the source image [60].

There are simpler methods that only normalize the image just by setting the mean and variance of source images to template images or in one study [61] it first converts to YUV color space and then sets mean to 0 and variance to 1.

#### Integral transformation methods

In histopathology images, different stains have different spectral properties which results in distinct color appearances. Integral transformation based methods incorporate information about the expected staining colors and their distribution. Using this information, Integral transformation methods establish a single transformation that can be applied to all the pixels in an image. By applying a unified transformation to all pixels in the image, these methods ensure that the normalized images maintain the underlying structural information. Furthermore, compared with separate transformation based methods, these methods minimize the risk of introducing artifacts and ensure that the color normalization process yields consistent results.

This transformation is often estimated using color deconvolution (CD) techniques. Color deconvolution aims to separate the mixed color information in stained tissue images into their constituent stain components. Various approaches have been proposed based on this approach. Vahadane et al. proposed a structure-preserving color normalization (SPCN) method that combines sparse non-negative matrix factorization (SNMF) for stain separation and a structure-preserving color normalization technique [15]. In SPCN, the color appearance and stain density maps of both the source and target images are estimated. The stain density map of the source image is scaled and combined with the color appearance of the target image to generate the normalized source image.

An improvement of the Vahadane’s method [15] proposed in [62]. The goal of this method is to estimate the stain representation and concentration in histological images to achieve better normalization results. The method described utilizes a sparsity parameter and stain representation matrix to normalize histological images. It estimates the sparsity parameter using a fuzzy method and initializes the stain representation matrix using a histogram. The stain representation is then estimated by minimizing the difference between input optical densities and the product of the matrix and a stain concentration map. The proposed method has a limitation in accurately estimating the stain representation matrix due to the influence of highly stained histological regions, such as stroma, red blood cells, and blood vessels. These regions, which show intense eosin staining, can negatively impact the normalization results.

In a related study [63], spectral matching techniques by employing non-negative matrix factorization (NMF) and its variations were explored. In this proposed technique, fuzzy theory and the Cuckoo search (CS) algorithm [64] were used to estimate stain representation and concentration. The proposed normalization method involves three essential steps. In the first step, the sparsity parameter was estimated. Following this, the stain color appearance matrix is initialized. Finally, the stain color appearance matrix is estimated by effectively separating and quantifying the staining components present in the image. Although spectral matching methods preserve the colors and textures present in histological images better than histogram matching and color transfer, one notable drawback of this method is its high computational cost. To estimate the parameters of the fuzzy function, the differential evolution (DE) and CS algorithms were used in studies [62] and [63], respectively. A comprehensive study was conducted to evaluate the impact of the algorithm on the final outcome [65]. The researchers found that although the choice of algorithm had little effect on the results, among those studied, the particle swarm optimization (PSO) algorithm [66] was the most appropriate for a population size of 250 individuals. In contrast, the metric used for the final estimation of the sparsity parameter had a significant impact on the results. Ultimately, the ​$l_{\varepsilon }^{0}$ metric produced the most suitable outcome for estimating the sparsity parameter.

However, it was discussed in [67] that mapping source color vector to a narrower target domain decreases the diversity of images and reduces the generalizability of the models. They proposed an alternative approach based on stain augmentation. Here, instead of replacing the source domain with the target stain intensities, they used mix-up augmentation and mixed the target and source domains using randomly interpolated stain color matrices.

Some color artifacts can occur with Vahadane’s method [15]; to address this limitation, an adaptive color deconvolution (ACD) model was presented in [68]. The ACD algorithm employs integral optimization techniques to estimate the parameters for both the hematoxylin and eosin stains simultaneously. By jointly estimating the parameters, ACD aims to capture the inherent relationships between the stain components more effectively and deliver robust normalization. ACD takes into account the prior knowledge of H&E stained slides, overall intensity, and proportion of stains. This consideration helps prevent color artifacts and preserves the key structural features of the tissue.

Considering that NMF does not have a closed-form solution, in the methods that proceed based on this, the solution is calculated numerically. Also, in these models, when three or more stains appear in the slides, the results will be inconsistent. To address this problem, the Macenko’s method [13] based on singular value decomposition (SVD) was introduced. It involves several steps: First, the RGB image is converted to optical density (OD) space. Then, pixels with low intensity are removed. The OD matrix undergoes SVD, and the two largest singular values are used to construct a plane in the OD space. All data points are projected onto this plane and normalized. The angle of each point to the SVD directions is calculated to measure its deviation from the staining vectors. To eliminate noise, percentile values are used instead of maximum and minimum angles. Finally, the extreme values are converted back to the OD space, resulting in the optical stain vector. This method is faster than the method based on NMF, also a pipeline based on pixel sampling and distributed implementation provided in [69] with the aim of reducing time to slide normalization; they performed their pipeline on Macenko’s [13] model with optimized multi-core implementation of the SVD and reduced the execution time.

Another normalization method was conducted based on Macenko’s model, but with stain separation achieved by removing the background and integrating structural information through segmentation of nuclei and stroma regions. Unlike Macenko’s method, which relies on a one-time stain estimation from the entire image, SCAN [70] iteratively refines the stain vectors based on OD values from segmented nuclei and stroma. This enables more biologically meaningful color deconvolution and better preservation of tissue morphology. Furthermore, instead of numerically inverting the stain matrix for normalization—which can violate the physical constraints of the Beer–Lambert law and yield negative densities—SCAN estimates stain densities in a robust and physically plausible manner.

In general, in methods based on NMF or SVD, the basis of the source color vectors is replaced with those from the reference, while the source and reference data may be different in the origin and size of the query basis wedge. Also in some models like Macenko’s [13] model illumination correction wasn’t implemented. To address this limitation Another method based on color deconvolution was suggested in [71]. Their approach addressed the normalization problem in three steps using the geometry of color vector space. They first applied Illumination correction. Then, they applied Stain Color Vector Correction adapted from Macenko’s [13] work. They used SVD to extract stain color vectors and selected the two vectors corresponding to the two largest eigenvalues as stain vectors. Finally, Stain quantity correction is done after obtaining the color histogram by adjusting the first percentile of the source to the first percentile of the reference and also adjusting the 99th percentile of the source to the 99th percentile of the reference. The proposed method tackles variations in illumination, stain chemical, and stain quantity by utilizing the geometry of the color vector space in a unified framework. In [72] illumination correction was considered and the accuracy of the analysis was enhanced by estimating illumination factors for each color channel separately. The method further refines the illumination map using Gamma correction [68], allowing for better adaptation to different lighting conditions. The final step involves recombining the normalized hematoxylin and eosin components to reconstruct the original image. This technique stands out for its precise color measurement and robust illumination adjustment.

The super Gaussian (SG) Bayesian framework was used to estimate the color-vector matrix and stain concentrations in [73]. The SG priors are instrumental in effectively reducing noise while preserving the tissue structure without excessively smoothing the edges. Finally, the estimated color-vector matrix and concentrations are obtained by automatically adjusting the parameters using the Kullback-Leibler (KL) divergence during the inference procedure. These estimates provide information about the different stains in the image and their respective concentrations. The information provided by this stain separation can then be used for the normalization problem. The use of prior on the color has the advantage of reducing the impact of noise and artifacts. Nevertheless, it can constrain adaptability to various color distributions. To overcome this issue, Bayesian framework that solves color deconvolution as a dictionary learning problem with sparse concentrations was introduced in [74]. The proposed method relies on the K-singular value decomposition (K-SVD) algorithm. The paper outlines two Bayesian inference approaches for color deconvolution: variational and empirical Bayes. Both approaches offer automated estimation of parameters, eliminating the need for manual intervention. The authors employ a hierarchical prior on stain concentrations, promoting sparsity the same as the Laplacian prior. This hierarchical prior simplifies the Bayesian inference process, enabling efficient estimation of stain concentrations.

In these methods, mostly independent color channels are assumed and the multimodal nature of each color channel is not considered during transformation. Pixels of images were clustered to stain colors with a mixture of multivariate skew-normal distributions in [75] to capture multimodality and correlation of colors. Also, they used Markov random field (MRF) to capture spatial correlation between pixels. After estimating the parameters of the models of source and reference images, color transformation was performed based on these parameters.

By incorporating information about expected staining colors and their distribution, integral transformation-based methods in stain normalization aim to address the distinct color appearances resulting from different stains in histopathology images. By utilizing a unified transformation across all pixels, these methods ensure consistent color adjustments while maintaining the relative differences in brightness and contrast within localized regions. the normalized images by these methods appear visually more natural and coherent.

#### Separated transformation

These methods aim to normalize the color appearance of histological images by transforming the stains separately. Separate transformation methods involve applying specific transformations to each stain separately to achieve color normalization.

In 2014, a color deconvolution approach was proposed in [76] based on nonlinear mapping to transfer color from a source to a target image using staining vector analysis. Pixels belonging to the same stain are extracted, and an estimation of a staining vector is obtained by analyzing the pixel distributions of the stains. A non-linear mapping is then created to transfer the color of different types of pixels to the template image, utilizing the staining vector. A nonlinear mapping approach to stain normalization in digital histopathology images using image-specific color deconvolution offers several advantages. Firstly, it effectively reduces the differences in color and brightness of digital pathology images, improving the performance of computer-aided diagnostic systems. It can be combined with color augmentation to further improve model performance. The approach employed utilizes a supervised linear mapping and classification technique for identifying the stain matrix. However, it may not yield satisfactory results when confronted with source images that have low contrast. Furthermore, this method is unable to retain all the biological information present in the source image.

In 2017, a method was proposed in [77] to reduce the inter-image variability by maintaining the color difference in the textural structures of the image. First, HSV colors are divided into ten clusters and each color cluster is assigned to a texture category in the form of an interactive interface. Finally, the colors will have a texture meaning this time, a four-channel data structure will be created. And each channel is associated with a tissue structure. Finally, color normalization is achieved by mapping pixels to target colors associated with each tissue element, determined using an average from a test image set.

In another investigation, a new normalization method was proposed in 2023 [53], where the process initiates by converting color patch images from digital slides to grayscale, serving as input for the score-based diffusion (SBD) model to generate normalized images. An overlapped moving window patch strategy is implemented to avoid grid artifacts, using overlapping patches to ensure image consistency. To prevent color misclassification between hematoxylin and eosin, the authors apply a stain separation technique using SNMF. The SBD model then colorizes the image, correcting for variations in staining intensity and distribution. Finally, the separately processed stain channels are recombined, mapping intensity values back to color, resulting in an image that emulates a traditionally stained slide.

### Hybrid frameworks: integrating non-deep and deep learning

Conventional models as well as deep learning models had their strengths and weaknesses. Proposing a model that could use both of these tools could take advantage of the strengths of each.

A color standardization module was introduced in [78], referred to as the stain standardization capsule (SSC). This method is based on the philosophy of traditional models using stain separation with a linear interaction between OD space and stain space, while leveraging the advantages of deep learning models. The deep learning part of SSC is based on a capsule network, in which, instead of scalars, neurons take vectors as input and output. The corresponding dynamic routing algorithm can learn and generate uniform stain separation outputs for histopathological images with various color appearances, without the need for manually selected template images. The module is lightweight and can be jointly trained with the application-driven Convolutional neural network (CNN) model [79], providing uniform input tensors for CNN in forward propagation. It employs a modified sparsity routing algorithm to select the most suitable stain separation parameters for each image, based on the pixel-wise and channel-wise sparsity measures of the stain candidates. Additionally, a reconstructed layer and loss function were employed to enhance generalizability.

LStainNorm [80], another hybrid model, is conducted similarly to Reinhard’s model [14] in that it reconstructs the image to have the same color statistics as the target. However, instead of using the mean and standard deviation of the target data, the statistics used for normalization consist of two parts: one fixed part obtained from the target data, and another trainable part learned by the deep learning model. The target images are selected using a random N-patch sampling strategy that automates the process and eliminates the need to manually identify a suitable reference. The use of different color spaces was previously seen in the RandStainNA++ [56]. In this study, unlike the earlier model that randomly selected a color space each time, all color spaces are selected for normalization, and the final result is obtained by combining all these RGB-normalized images through a self-attention layer [81]. This model is accompanied by a downstream analysis for predicting biological features such as calcification, where all parameters of the model are trained jointly based on the overall cost function. Furthermore, incorporating biological context and structural understanding of tissue morphology can enhance the normalization process.

### Signal processing-based

Signal processing is an important field of engineering science. One of the techniques widely used in this field is the Fourier transform, which converts a signal from the time domain to the frequency domain. This transformation provides valuable information that represents the original signal [82]. The Fourier transform has many applications, including in computer vision and image segmentation [83,84].

The first study in the field of normalization conducted in this category was proposed in [85]. In this study, normalization and augmentation of pathology images began by transferring the images from the color space to the frequency domain. They were inspired by this technique because many medical signals originate from frequency information. Image channels in the color space represent color, but by applying a Fourier transform and converting the image to the frequency domain, each channel represents frequency. Analysis of the frequency space of the images showed that the main information related to the coloring style and illumination is usually recorded in the low-frequency components, while noise, color variations, and quality-related aspects are often recorded in the mid-to-high frequency spectrum. As a result, manipulation of low frequencies is useful for normalization and color stability. The normalization process in this study is performed by aligning the amplitudes in the frequency space. Both the source and target images are transformed into frequency space using a Fast Fourier Transform (FFT), and then their arithmetic mean is calculated using a mask extracted with a deep learning model. The inverse Fourier transform of the result of these steps produces a normalized image in the color space. This mask determines whether each element participates in the normalization process and regulates the relative influence of the source and target on the final outcome. The mask was trained using unsupervised deep learning with the aim that the normalized image not only retains the composite details of the source image but also incorporates the color style information of the target image. In this study, data augmentation is also performed in the Fourier space using the arithmetic mean between Gaussian noise and the Fourier transform of the source image.

## Results

In general, we conducted three analyses: (1) evaluation of normalization methods using common metrics; (2) assessment of the models’ ability to remove scanner-related; and (3) evaluation of their capability to preserve biological information. In the first analysis, we implemented normalization methods on the SCAN data and evaluated the results using the common metrics mentioned in the Materials and Methods section. MITOS-ATYPIA-14 data was used to examine the ability of models to remove scanner effects. TCGA data was utilized for assessing biological information retention. Deep learning-based methods like CycleGAN [29] and StainNet [31] used pre-trained weights from original publications.

### Structural similarity evaluation

**Fig. 6:**
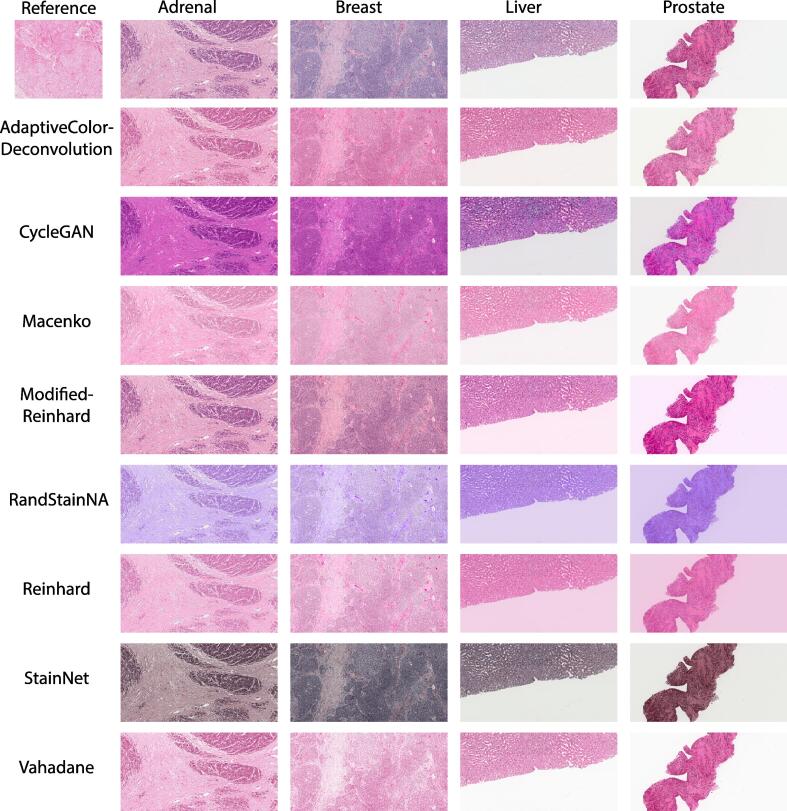
Sample source images and correspond normalized images with different normalization method at resolution 10x.

The normalization algorithms were applied to the SCAN data and image similarity was evaluated using metrics. As shown in table 1 and 2, regardless of the resolution and tissue of the images, the RandstainNA [54] and Reinhard [14] methods showed minimal color variation, indicating good color similarity. Considering that there is a different combination of tissue and background in each image, this metric cannot be a suitable criterion alone. The background color in normalized images influenced the NMI values. For liver images, the two methods added more background color, which may explain the low standard deviation of NMI values observed for these methods, as shown in Fig. 6.

The SSIM measurement, which evaluates the structural similarity, was highest for ModifiedReinhard [55] method, followed closely by the Vahadane [15] and AdaptiveColorDeconvolution [68] methods (see Table 1, Table 2).

**Table 1:** Comparison between source and normalized images at resolution 10x.

Tissue	Algorithm	NMI mean	NMI sd	SSIM	PSNR	RMSE	MSSSIM	LPIPS	FSIM
Adrenal	AdaptiveColorDeconvolution [68]	0.751	0.055	0.939	49.612	**0.003**	0.967	0.784	**0.82**
	CycleGAN [29]	0.651	0.054	0.753	41.667	0.008	0.914	0.452	0.718
	Macenko [13]	0.795	0.033	0.847	44.495	0.006	0.901	0.61	0.813
	ModifiedReinhard [55]	0.744	0.068	**0.948**	**51.908**	**0.003**	**0.981**	**0.867**	0.812
	RandstainNA [54]	0.795	**0.018**	0.918	42.893	0.007	0.859	0.688	0.673
	Reinhard [14]	0.796	**0.018**	0.907	45.859	0.005	0.932	0.705	0.809
	StainNet [31]	0.582	0.087	0.892	39.365	0.01	0.925	0.367	0.815
	Vahadane [15]	0.808	0.052	0.942	46.54	0.005	0.963	0.733	0.813
Breast	AdaptiveColorDeconvolution [68]	0.777	0.046	0.943	46.189	**0.004**	0.954	0.695	**0.824**
	CycleGAN [29]	0.632	0.053	0.749	38.925	0.01	0.911	0.342	0.7
	Macenko [13]	0.799	0.028	0.895	44.536	0.006	0.919	0.574	0.826
	ModifiedReinhard [55]	0.781	0.07	**0.949**	**47.847**	**0.004**	**0.979**	**0.746**	0.817
	RandstainNA [54]	0.795	**0.009**	0.937	44.099	0.006	0.883	0.66	0.73
	Reinhard [14]	0.795	**0.009**	0.936	44.864	0.006	0.944	0.622	0.819
	StainNet [31]	0.61	0.098	0.892	40.104	0.01	0.929	0.501	0.817
	Vahadane [15]	0.855	0.028	0.938	44.221	0.006	0.944	0.696	0.814
Liver	AdaptiveColorDeconvolution [68]	0.82	0.118	0.942	46.68	0.005	0.969	0.709	0.783
	CycleGAN	0.746	0.134	0.874	46.636	0.005	0.938	0.629	0.714
	Macenko [13]	0.859	0.099	0.893	42.846	0.007	0.931	0.615	0.792
	ModifiedReinhard [55]	0.785	0.166	**0.965**	**52.292**	**0.002**	**0.985**	**0.86**	0.786
	RandstainNA [54]	0.837	**0.074**	0.87	40.843	0.009	0.871	0.581	0.662
	Reinhard [14]	0.839	**0.075**	0.866	42.552	0.008	0.906	0.564	0.764
	StainNet [31]	0.645	0.221	0.908	39.808	0.01	0.952	0.541	0.783
	Vahadane [15]	0.819	0.155	0.963	49.022	0.003	0.979	0.843	**0.795**
Prostate	AdaptiveColorDeconvolution [68]	0.88	0.11	0.945	45.752	0.006	0.97	0.735	0.758
	CycleGAN [29]	0.797	0.14	0.913	46.869	0.004	0.951	0.728	0.679
	Macenko [13]	0.916	0.092	0.879	41.718	0.009	0.915	0.671	0.756
	ModifiedReinhard [55]	0.838	0.184	**0.975**	**51.657**	**0.003**	**0.988**	**0.873**	0.755
	RandstainNA [54]	0.869	**0.069**	0.875	39.821	0.01	0.864	0.614	0.638
	Reinhard [14]	0.871	0.07	0.877	41.096	0.009	0.905	0.585	0.713
	StainNet [31]	0.724	0.248	0.904	40.777	0.009	0.953	0.653	0.732
	Vahadane [15]	0.868	0.176	0.974	49.235	**0.003**	0.983	0.838	**0.768**

**Table 2:** Comparison between source and normalized images at resolution 20x.

Tissue	Algorithm	NMI	NM	SSIM	PSNR	RMSE
		mean	sd			
Adrenal	AdaptiveColorDeconvolution [68]	0.764	0.038	0.963	49.518	**0.003**
	CycleGAN [29]	0.675	0.045	0.85	43.135	0.006
	Macenko [13]	0.805	0.033	0.877	44.851	0.006
	ModifiedReinhard [55]	0.755	0.057	**0.971**	**51.593**	**0.003**
	RandstainNA [54]	0.797	**0.013**	0.931	43.157	0.006
	Reinhard [14]	0.786	**0.014**	0.944	46.973	0.004
	StainNet [31]	0.591	0.073	0.905	39.588	0.01
	Vahadane [15]	0.804	0.047	0.966	47.36	0.004
Breast	AdaptiveColorDeconvolution	0.794	0.045	0.967	46.525	**0.004**
	CycleGAN [29]	0.686	0.05	0.869	41.361	0.008
	Macenko [13]	0.814	0.033	0.928	44.934	0.005
	ModifiedReinhard [55]	0.802	0.057	**0.971**	**47.803**	**0.004**
	RandstainNA [54]	0.806	**0.02**	0.957	44.293	0.006
	Reinhard [14]	0.796	0.023	0.961	45.007	0.005
	StainNet [31]	0.64	0.085	0.911	40.468	0.009
	Vahadane [15]	0.861	0.033	0.964	44.912	0.006
Liver	AdaptiveColorDeconvolution [68]	0.674	0.019	0.937	45.705	0.006
	CycleGAN [29]	0.59	0.048	0.872	46.042	0.005
	Macenko [13]	0.739	0.018	0.863	41.099	0.009
	ModifiedReinhard [55]	0.582	0.072	**0.969**	**52.752**	**0.002**
	RandstainNA [54]	0.759	**0.008**	0.877	38.814	0.011
	Reinhard [14]	0.744	**0.008**	0.894	41.241	0.009
	StainNet [31]	0.385	0.078	0.878	38.143	0.012
	Vahadane [15]	0.652	0.072	0.96	46.242	0.005
Prostate	AdaptiveColorDeconvolution [68]	0.7	0.041	0.914	43.489	0.007
	CycleGAN [29]	0.595	0.068	0.834	44.58	0.006
	Macenko [13]	0.775	0.027	0.797	39.037	0.012
	ModifiedReinhard [55]	0.599	0.122	**0.975**	**51.88**	**0.003**
	RandstainNA [54]	0.781	**0.012**	0.84	38.403	0.012
	Reinhard [14]	0.769	0.014	0.859	40.052	0.011
	StainNet [31]	0.408	0.128	0.833	38.32	0.012
	Vahadane [15]	0.681	0.109	0.96	44.03	0.006
Colon	AdaptiveColorDeconvolution [68]	0.736	0.042	0.922	44.253	0.006
	CycleGAN [29]	0.628	0.055	0.845	46.856	0.004
	Macenko [13]	0.808	0.035	0.782	38.422	0.012
	ModifiedReinhard [55]	0.64	0.073	**0.967**	**53.729**	**0.002**
	RandstainNA [54]	0.799	**0.016**	0.851	38.332	0.011
	Reinhard [14]	0.787	0.017	0.871	40.726	0.009
	StainNet [31]	0.456	0.087	0.865	38.368	0.011
	Vahadane [15]	0.719	0.056	0.954	44.695	0.005

**Fig. 7:**
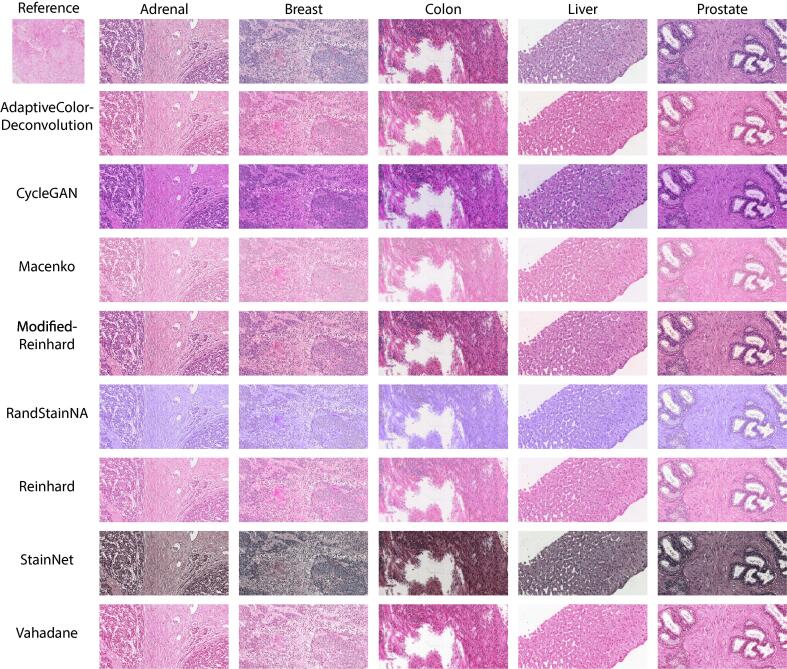
Sample source images and correspond normalized images with different normalization method at resolution 20x.

**Fig. 8:**
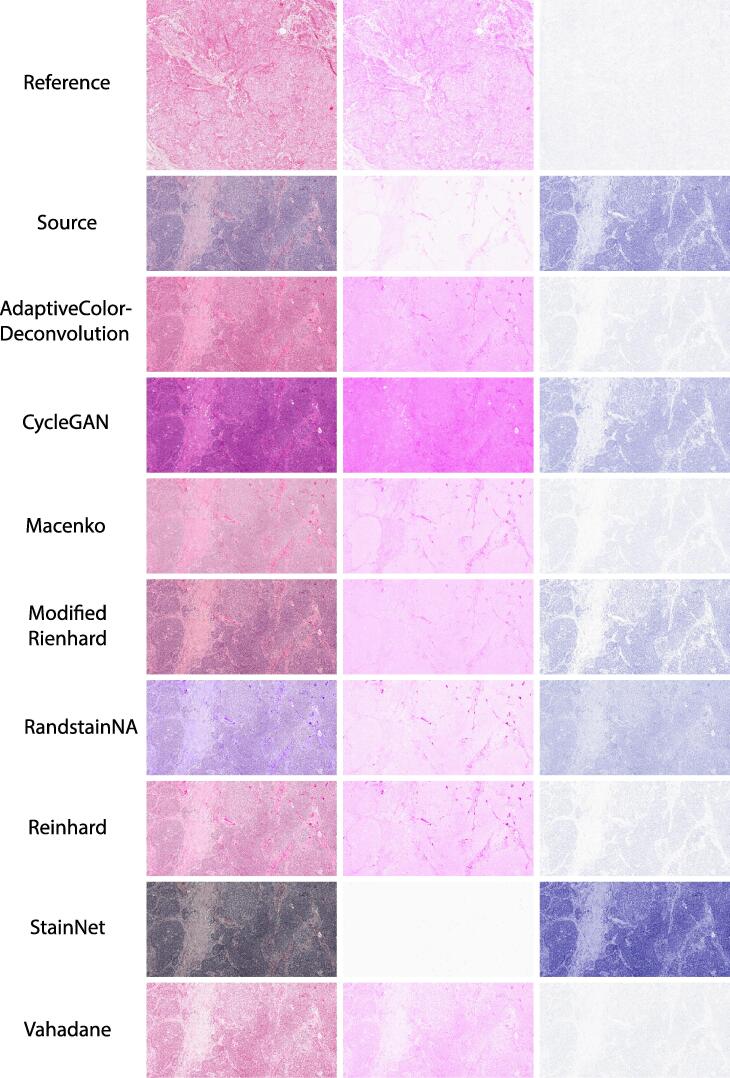
The first row indicates that the Reference image was used in the normalization method. The second row is a sampled source image and corresponding to eosin and hematoxylin images. In the first column original images are shown and the second and third (from left) are eosin and hematoxylin images, respectively. Source and normalized images are at a resolution of 10x.

**Fig. 9:**
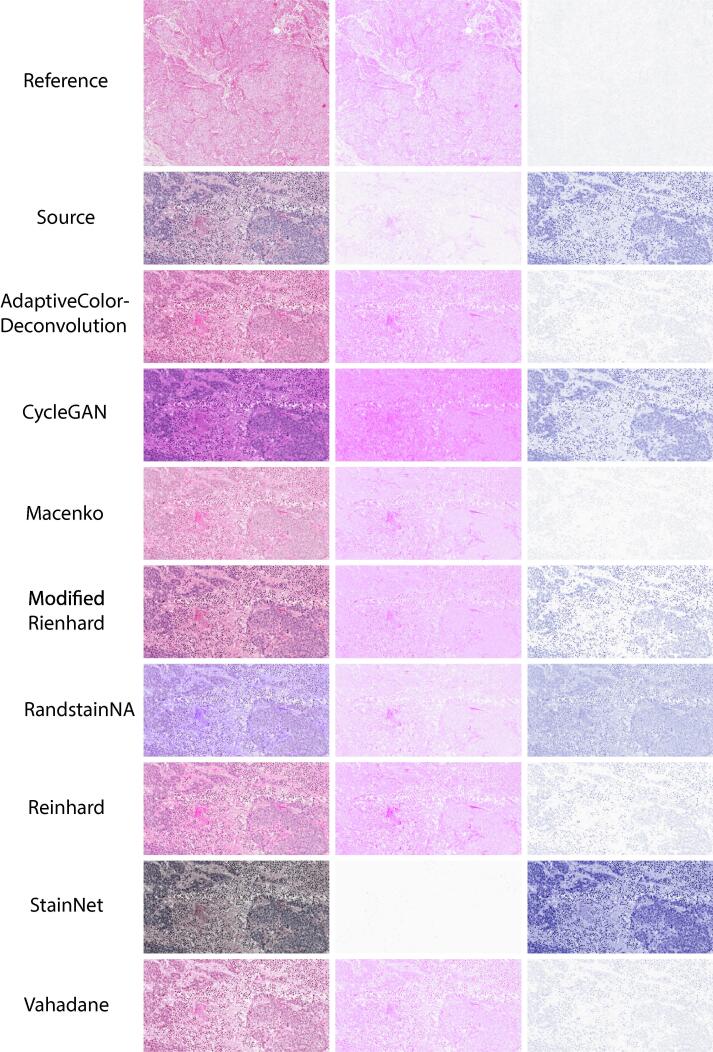
The first row indicates Reference image was used in the normalization method. The second row is a sampled source image and corresponds to eosin and hematoxylin images. In the first column original images are shown and the second and third (from left) are eosin and hematoxylin images, respectively. Source and normalized images are at a resolution of 20x.

The RMSE metric measures the difference between images and is also affected by color changes. The PSNR metric, which is derived from RMSE, along with RMSE itself, favored the AdaptiveColorDeconvolution [68] and ModifiedReinhard [55] algorithms, indicating better retention of source image information, including color. As shown in Fig. 6, Fig. 7, the color similarity between the normalized image and the source image is higher for the ModifiedReinhard [55] method compared to the AdaptiveColorDeconvolution [68] method. This trend is also observable in Fig. 8, Fig. 9, where the similarity is visible in both the eosin and hematoxylin color channels.

According to Fig. 2, Fig. 4, Reinhard [14] preserved eosin better but underperformed with hematoxylin colors. As shown in Fig. 3, Fig. 5, this may be influenced by the color composition of the reference image. See also Table 3, Table 4 for quantitative comparisons.

**Table 3:** Summary of NMI values of eosin and hematoxylin stains of images at resolution 10x.

Tissue	Algorithm	Eosin Stain		Hematoxylin Stain	
		Mean	Sd	Mean	Sd
Adrenal	AdaptiveColorDeconvolution [68]	0.0397294	0.038423	0.011557	0.007889
	CycleGAN [29]	0.0358104	0.008143	0.061942	0.015904
	Macenko [13]	0.1483548	0.060353	0.000795	0.003557
	ModifiedReinhard [55]	0.0172787	0.001473	0.017259	0.015164
	RandstainNA [54]	0.1710551	0.109475	0.184969	0.055092
	Reinhard [14]	0.165419	0.110601	0.011257	0.006977
	StainNet [31]	0	0	0.020115	0.005052
	Vahadane [15]	0.0177035	0.010332	0.004739	0.005592
Breast	AdaptiveColorDeconvolution [68]	0.0924093	0.043647	0.013245	0.014509
	CycleGAN [29]	0.0353511	0.006397	0.079118	0.050722
	Macenko [13]	0.0967209	0.031933	0.01256	0.020694
	ModifiedReinhard [55]	0.0521677	0.061616	0.025685	0.036548
	RandstainNA [54]	0.0103875	0.000497	0.152499	0.022522
	Reinhard [14]	0.0169410	0.009655	0.01525	0.005161
	StainNet [31]	0	0	0.024151	0.009885
	Vahadane [15]	0.0533307	0.035943	0.010416	0.010818
Liver	AdaptiveColorDeconvolution [68]	0.1086972	0.111092	0.018758	0.005378
	CycleGAN [29]	0.0245577	0.033406	0.06381	0.04116
	Macenko [13]	0.1163941	0.103801	0.01132	0.007221
	ModifiedReinhard [55]	0.0268596	0.034095	0.012426	0.018242
	RandstainNA [54]	0.3157004	0.15912	0.270586	0.118674
	Reinhard [14]	0.3039514	0.147591	0.026411	0.005803
	StainNet [31]	0.0006224	0.001495	0.027593	0.031282
	Vahadane [15]	0.0408159	0.056025	0.009182	0.018358
Prostate	AdaptiveColorDeconvolution [68]	0.0671949	0.099305	0.014297	0.00314
	CycleGAN [29]	0.0114898	0.01671	0.031505	0.01806
	Macenko [13]	0.0767676	0.110012	0.013563	0.005084
	ModifiedReinhard [55]	0.0141585	0.011514	0.000833	0.001481
	RandstainNA [54]	0.258897	0.177533	0.260959	0.142117
	Reinhard [14]	0.2499836	0.160319	0.033621	0.007219
	StainNet [31]	0.0002674	0.000778	0.011598	0.01042
	Vahadane [15]	0.0113069	0.016181	0.001684	0.001361

**Table 4:** Summary of NMI values of eosin and hematoxylin stains of images at resolution 20x.

Tissue	Algorithm	Eosin Stain		Hematoxylin Stain	
		Mean	Sd	Mean	Sd
Adrenal	AdaptiveColorDeconvolution [68]	0.069904	0.069601	0.007426	0.00494
	CycleGAN [29]	0.029685	0.006395	0.042377	0.019288
	Macenko [13]	0.12938	0.057211	0.000562	0.002697
	ModifiedReinhard [55]	0.026335	0.022424	0.008004	0.010595
	RandstainNA [54]	0.220922	0.110415	0.22324	0.070183
	Reinhard [14]	0.165317	0.116994	0.002471	0.003394
	StainNet [31]	0	0	0.020436	0.00815
	Vahadane [15]	0.032214	0.039367	0.005215	0.005944
Breast	AdaptiveColorDeconvolution [68]	0.109488	0.037855	0.008128	0.007509
	CycleGAN [29]	0.026195	0.005874	0.056999	0.031958
	Macenko [13]	0.118254	0.035614	0.00196	0.004789
	ModifiedReinhard [55]	0.03996	0.044712	0.00739	0.012876
	RandstainNA [54]	0.015445	0.019748	0.176072	0.035255
	Reinhard [14]	0.016457	0.001019	0.002728	0.002288
	StainNet [31]	0	0	0.021878	0.009417
	Vahadane [15]	0.05824	0.035696	0.007452	0.007845
Colon	AdaptiveColorDeconvolution [68]	0.216878	0.053252	0.009583	0.004811
	CycleGAN [29]	0.035775	0.009715	0.066304	0.027759
	Macenko [13]	0.17754	0.0374	0.000898	0.002677
	ModifiedReinhard [55]	0.022194	0.00213	0.028895	0.018745
	RandstainNA [54]	0.359833	0.055656	0.34395	0.046208
	Reinhard [14]	0.305284	0.052912	0.002555	0.002854
	StainNet [31]	0.000694	0.001224	0.027117	0.005939
	Vahadane [15]	0.0551	0.037873	0.008674	0.010203
Liver	AdaptiveColorDeconvolution [68]	0.285237	0.097905	0.02364	0.007898
	CycleGAN [29]	0.048502	0.023812	0.073457	0.030812
	Macenko [13]	0.260821	0.043765	0.001841	0.004466
	ModifiedReinhard [55]	0.04259	0.035082	0.039832	0.020001
	RandstainNA [54]	0.4267	0.111847	0.383319	0.094945
	Reinhard [14]	0.393187	0.135082	0.001219	0.001971
	StainNet [31]	0.002737	0.002582	0.034589	0.011621
	Vahadane [15]	0.075238	0.064865	0.008178	0.014236
	AdaptiveColorDeconvolution [68]	0.175263	0.10027	0.013403	0.006577
Prostate	CycleGAN [29]	0.041879	0.029707	0.092951	0.040228
	Macenko [13]	0.205283	0.079986	0.001164	0.003253
	ModifiedReinhard [55]	0.028433	0.026272	0.039478	0.035143
	RandstainNA [54]	0.323709	0.129424	0.355789	0.119771
	Reinhard [14]	0.280397	0.129218	0.000907	0.002186
	StainNet [31]	0.000209	0.000684	0.034354	0.01084
	Vahadane [15]	0.049533	0.035898	0.010477	0.017686

Vahadane [15] scored highest on the FSIM metric and performed comparably to ModifiedReinhard according to SSIM, PSNR, and RMSE for some tissues, Table 1, Table 2.

Overall, the two deep learning-based models, CycleGAN [29] and StainNet [31], often demonstrated lower similarity and performance compared to other methods. This was expected, as the pretrained weights provided by the original studies were used without further fine-tuning. These findings underscore the importance of retraining or fine-tuning such models on the target dataset when deploying them as stain normalization methods.

### Scanner type prediction

Images captured by different scanners are different in color. We used the deep learning model to evaluate the difference between these images. Considering that the data in MITOS-ATYPIA-14 includes digital images of breast tissue that were recorded with two scanners, they were suitable for evaluation in this section. We first used a deep learning model on these images with the aim of predicting the scanner. As expected, the images can be distinguished with high accuracy. Considering that the purpose of normalization is to bring the images closer to each other in color, it is expected that the images of the two scanners will be similar and less distinguishable after normalization. We normalized the images using different normalization methods. Then, we used a deep learning structure to train and predict the scanner on the normalized images. A pre-trained VGG model with two additional fully connected layers was used as the deep learning model, and the images were tiled to 224 × 224 in order to be fed into this model.

It's important to note that the CycleGAN [29] and StainNet [31] models, being deep learning models that have already been trained on the same training data and using the same weights, may not provide a fair comparison with other models on this data. However, we have included the results of these two models in the table. Additionally, our dataset contains images of two resolutions, 20x and 40x, necessitating a comprehensive analysis on both resolutions to ensure a thorough understanding of the data. Macenko [13] has best result for both of resolutions, see Table 5.

**Table 5:** Result of the scanner prediction model of normalized images.

Algorithm	Accuracy	
	20x	40x
AdaptiveColorDeconvolution [68]	0.875	0.927
CycleGAN [29]	0.856	**0.787**
Macenko [13]	**0.848**	**0.874**
ModifiedReinhard [55]	0.954	0.936
RandstainNA [54]	0.956	0.918
Reinhard [14]	0.950	0.934
StainNet [31]	0.957	0.945
Vahadane [15]	0.855	0.881

### Cancer subtype prediction

One of the important aspects of using stain normalization models in the pre-processing of digital image data is to train deep learning models to predict biological information, so these normalization models should preserve biological information during color normalization. To evaluate these models in preserving biology information, we used the TCGA dataset and a deep learning model to predict the cancer subtype of images. The deep learning model used in this part was based on pretrained VGG16. And we have used transfer learning technique. According to the input of the VGG16 model, we tiled the images with dimensions of 224 x 224. Extracted only the tiles that made up 90 % of the texture, and excluded the others from the study. Eighty percent of the data was used to train and validation, and test was performed of 20 % of the data. The test set was the same for all normalization methods. Images are from 6 subtypes of breast cancer and almost an equal number of WSIs have been taken from each cancer subtype.

The result shows that Vahadane [15] was assigned the best result, and CycleGAN [29] has minimum accuracy, see Table 6. It should be noted that we have not done any fine-tuning on the two methods based on deep learning, CycleGAN [29] and StainNet [31].

**Table 6:** Result of the cancer subtype prediction model of normalized images.

Algorithm	Accuracy
AdaptiveColorDeconvolution [68]	0.771
CycleGAN [29]	0.691
Macenko [13]	0.747
ModifiedReinhard [55]	0.802
RandstainNA [54]	0.706
Reinhard [14]	0.751
StainNet [31]	0.783
Vahadane [15]	**0.820**

## Discussion and future directions

Pathologists use the images according to the features in them, such as staining patterns or the size of the nucleus, to diagnose and classify the treatment [86,87]. Although manual analysis methods are still used, the extraction of information in this way is time-consuming and depends on the skills of the pathologists [88]. Automatic algorithms are used to overcome the limitations of manual analysis, and one of the most widely used methods for automatic analysis is the use of deep learning. Many studies have tried to use deep learning to predict biological information including, cancer subtypes [89,90] or type of gene modifications [91,92] from pathological images. The test results of trained models on unseen data indicate a decrease in performance [93,94]. One of the main reasons for this performance drop is the color variation between images from different datasets. Normalization models with the aim of adjusting the difference in the color of images obtained from a source other than biological information, improve the performance of models on dataset images independent of the dataset [94]. Considering that image data is increasing day by day, automatic preprocessing and normalization are necessary, so although supervised algorithms may have good results, being fully automatic has its own importance. In addition to enhancing deep learning models, researches showed that applying the SCAN [70] normalization technique led to improved color consistency, faster analysis, and better agreement among pathologists [95]. In a similar study, it was observed that applying Vahadane’s normalization method to prostate pathology images led to a 5–6 % increase in diagnostic accuracy by pathologists and a reduction in diagnosis time by up to 29 % [96].

In a study, H&E histopathology images of melanoma biopsies from 5 different hospitals were collected. In this study, the researchers defined 4 tasks: predicting the patient's age, predicting the type of scanner used to take the image, predicting the Slide origin (the hospital where the image was taken) and slide preparation date. Considering that they achieved 100 % accuracy in the type scanner prediction task and almost 97.9 % accuracy in the slide source prediction test, obviously, the slides from different scanners and different sources are different in color, one of the goals of normalization models is to adjust these differences [97].

In this article, we have attempted to evaluate color normalization models by applying several approaches on three datasets and comparing their performance. Unlike most frameworks that focus on a single evaluation criterion (e.g., visual similarity or classification accuracy), our approach evaluates normalization methods using three distinct tasks: assessment with traditional metrics, scanner prediction, and cancer subtype prediction. We assessed structural and non-structural similarities using six traditional metrics between the original and normalized images generated by several algorithms; this evaluation was performed on data from multiple tissue types.

We utilized the MITOS-ATYPIA-14 dataset to assess the reduction of scanner-induced color differences (with images from two scanners) and our inclusion of images at two resolutions (20x and 40x) adds further depth and thoroughness to the evaluation. Furthermore, given that deep learning models are capable of capturing complex and non-linear patterns, the scanner prediction task enables a more accurate detection of scanner-induced color variations, offering a more sensitive and informative measure than traditional metrics alone. This ensures the robustness of the methods across varying imaging conditions—a critical factor often overlooked. The TCGA dataset was used to evaluate the preservation of biological information (six breast cancer subtypes). This framework provides a more robust and balanced assessment than the single-task evaluations commonly found in the literature. Our evaluation models were based on a tile-based approach. However, since WSI-level assessments are more reliable for evaluating the capture of biological information, we recommend such approaches. This remains a limitation of our study.

The methods compared in this study focused solely on stain normalization. According to the results reported in [49], stain color augmentation can provide equal or even superior improvements in model generalization compared to normalization alone. However, more recent work demonstrates that, in certain contexts such as coeliac disease classification from duodenal biopsies, stain normalization yields greater generalizability than stain jittering, particularly when test data originates from different scanners and laboratories [98]. These apparently contradictory findings highlight that the optimal choice between stain normalization and augmentation is likely dependent on the specific dataset, task, and variability of data sources. Therefore, future studies should evaluate both approaches individually and in combination to determine the most effective method for their application.

On the first dataset, we examine the structural similarity and color variation before normalization and after normalization by different methods. What we observed was the superiority of ModifiedReinhard [55] in maintaining similarities.

In this study, we examined the differences in images resulting from the type of scanner. However, images acquired with the same scanner from different sources also differ from each other. Therefore, it is suggested that researchers check this issue before using a normalization model in their study. In examining the capability of normalization models in maintaining biology information, we limited ourselves to preserving information related to cancer subtype. It is recommended to check the preservation of the embedded biology information in more detailed images such as the type of mutations and the segmentation of different entities.

We used an image from the MITOS-ATYPIA-14 dataset as a reference to apply normalization methods on the SCAN dataset images. Considering that the reference image used is from breast tissue and the source images are from different tissues, it is recommended that researchers investigate this case further according to the tissue of their study and their data. Here, considering that, we wanted to use a single reference to be able to compare.

The results of normalization indicate that the selection of the reference image for the normalization algorithm is effective in the final result. In one of the studies, which was done in order to improve the segmentation of pathology images using normalization, researchers tried to train the deep learning model by selecting the normalization reference image in a non-deterministic way from some organs and normalized the image with all available references during the test and obtained the final result according to weighted average of result of each normalized image. Although they had used Macenko’s algorithm in this method, with this way of choosing the reference, their results were significantly improved [99]. It is suggested to do a similar process by randomly selecting a reference image from several datasets (one tissue) and using several normalization algorithms randomly.

However, beyond the question of how to select reference images lies another key consideration: how many should be used to minimize bias and improve generalizability. While relying on a single WSI can cause color bias by failing to represent dataset diversity, using multiple WSIs without a clear strategy may also introduce errors. To address this, a study [100] assessed the optimal number of reference images. Groups with different numbers of WSIs were randomly selected (with replacement), and Euclidean distances between stain vectors (via stain deconvolution) were calculated. The standard deviation (SD) of these distances quantified each group’s dispersion. Then, the SD ratio—SD of group with n + 1 images divided by that of group with n images—was computed. A sigmoid function was fitted to these ratios, and its derivative was analyzed. The point where the derivative neared zero indicated that adding more images no longer significantly reduced variability, defining the optimal number of reference WSIs. In this work, reference images were selected randomly. However, selecting reference WSIs based on structural or statistical characteristics—rather than random sampling—may lead to more representative choices. This strategy could reduce the computational burden of determining the optimal number of images while potentially improving normalization robustness.

## Conclusion

Histology images are a cornerstone of pathology, and allows automated analysis for disease diagnosis. However, because stain color varies between sources, the normalization method is designed to increase the accuracy of computer-aided diagnosis systems. Normalization methods can be supervised or unsupervised; with the increase in the use of pathology images, the necessity of using automatic normalization algorithms has increased. These methods can be divided into two general categories: deep learning-based and non-deep learning-based models. Algorithms based on deep learning, although they can perform well, need a lot of data and a lot of computation resources to train. We applied 8 normalization methods in this review on 3 datasets and compared the results. Among these methods, the ModifiedReinhard [55] algorithm has been one of the best in maintaining the similarity between the source images and corresponding normalized images, but it has not performed well in removing the color variation due to the scanner type. Vahadane [15] has well performance in maintaining similarity and also well performance in removing stain variation due to scanner type, also this method is best in preserving biological information of images. Macenko [13] has been the most successful non-deep learning-based algorithm in removing scanner-induced color variation, but it has not been at the top of the table in preserving biology information. Each algorithm has advantages and disadvantages, and we have reviewed some of them in this article. It is suggested to apply more checks on your research tissue and problem for the best algorithm for your study's data.

## Declaration of generative AI and AI-assisted technologies in the writing process

During the preparation of this work, the authors used ChatGPT to obtain a proper version of the text in some parts for English improvement. After using this tool, the authors review and edit the content as needed and take full responsibility for the content of the published article.

## Funding

This research did not receive any specific grant from funding agencies in the public, commercial, or not-for-profit sectors.

## Declaration of competing interest

The authors declare that they have no known competing financial interests or personal relationships that could have appeared to influence the work reported in this paper.
